# 7-(5-Methyl­sulfanyl-β-d-erythrofuran­osyl)-7*H*-pyrrolo­[2,3-*d*]pyrimidin-4-amine monohydrate (MT-tubercidin·H_2_O)

**DOI:** 10.1107/S1600536810020179

**Published:** 2010-06-18

**Authors:** Graeme J. Gainsford, Richard F. G. Fröhlich, Gary B. Evans

**Affiliations:** a Carbohydrate Chemistry Group, Industrial Research Limited, PO Box 31-310, Lower Hutt 5040, New Zealand

## Abstract

The title compound, C_12_H_16_N_4_O_3_S·H_2_O, which has potential as a possible anti­malarial drug, was studied when small deviations in melting points, for two differently aged preparations, were observed. The unexpected existence of a water mol­ecule of crystallization is considered to be the cause of this variation. The 7*H*-pyrrolo­[2,3-*d*]pyrimidine unit is very slightly puckered with a total puckering amplitude of 0.035 (2) Å; its mean plane makes an angle of 88.40 (12)° with the mean plane through the ribofuranosyl unit. In the crystal, the mol­ecules are bound by strong O—H⋯N and N—H⋯O hydrogen bonds, utilizing all available protons and linking mainly through the water of crystallization.

## Related literature

For details of the synthesis of and for background to the title compound, see: Riegelhaupt *et al.* (2010 ▶). For related structures, see: Seela *et al.* (2007 ▶); Abola & Sundaralingam (1973 ▶). For ring conformations, see: Cremer & Pople (1975 ▶). For hydrogen-bond motifs, see: Etter *et al.* (1990 ▶); Bernstein *et al.* (1995 ▶).
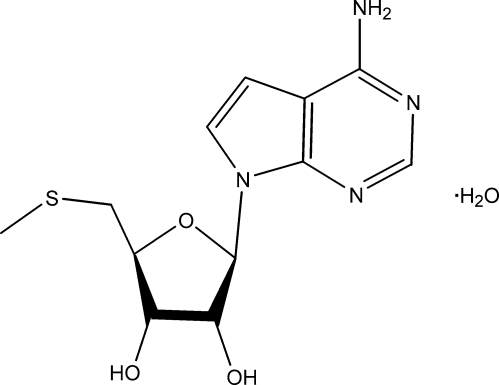


## Experimental

### 

#### Crystal data

C_12_H_16_N_4_O_3_S·H_2_O
*M* *_r_* = 314.36
Orthorhombic, 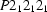

*a* = 4.790 (1) Å
*b* = 16.610 (3) Å
*c* = 18.020 (4) Å
*V* = 1433.7 (5) Å^3^
*Z* = 4
Cu *K*α radiation
μ = 2.22 mm^−1^
*T* = 100 K
0.50 × 0.02 × 0.02 mm

#### Data collection

Rigaku Spider diffractometer
Absorption correction: multi-scan (*ABSCOR*; Higashi, 1995 ▶) *T*_min_ = 0.712, *T*_max_ = 1.0
8013 measured reflections
2582 independent reflections
2422 reflections with *I* > 2σ(*I*)
*R*_int_ = 0.045

#### Refinement

*R*[*F* ^2^ > 2σ(*F* ^2^)] = 0.039
*wR*(*F* ^2^) = 0.091
*S* = 1.08
2582 reflections
209 parameters
2 restraints
H atoms treated by a mixture of independent and constrained refinement
Δρ_max_ = 0.29 e Å^−3^
Δρ_min_ = −0.33 e Å^−3^
Absolute structure: Flack (1983 ▶), 986 Friedel pairs
Flack parameter: 0.02 (2)

### 

Data collection: *CrystalClear* (Rigaku Americas, 2005 ▶); cell refinement: *FSProcess* in *PROCESS-AUTO* (Rigaku, 1998 ▶); data reduction: *FSProcess* in *PROCESS-AUTO*; program(s) used to solve structure: *SHELXS97* (Sheldrick, 2008 ▶); program(s) used to refine structure: *SHELXL97* (Sheldrick, 2008 ▶); molecular graphics: *ORTEP* in *WinGX* (Farrugia, 1999 ▶) and *Mercury* (Macrae *et al.*, 2006 ▶); software used to prepare material for publication: *SHELXL97* and *PLATON* (Spek, 2009 ▶).

## Supplementary Material

Crystal structure: contains datablocks global, I. DOI: 10.1107/S1600536810020179/kp2257sup1.cif

Structure factors: contains datablocks I. DOI: 10.1107/S1600536810020179/kp2257Isup2.hkl

Additional supplementary materials:  crystallographic information; 3D view; checkCIF report

## Figures and Tables

**Table 1:** Hydrogen-bond geometry (Å, °)

*D*—H⋯*A*	*D*—H	H⋯*A*	*D*⋯*A*	*D*—H⋯*A*
O2′—H2′*O*⋯N3^i^	0.79 (3)	1.99 (3)	2.776 (3)	173 (3)
O1*W*—H1*A*⋯O3′	0.82 (3)	1.96 (3)	2.748 (3)	162 (3)
O1*W*—H1*B*⋯O2′^ii^	0.80 (3)	2.07 (3)	2.848 (3)	162 (3)
O3′—H3′*O*⋯N1^iii^	0.84 (3)	1.94 (3)	2.766 (3)	166 (3)
N6—H6*A*⋯O1*W*^iv^	0.90 (3)	2.12 (3)	2.998 (3)	166 (3)
N6—H6*B*⋯O1*W*^v^	0.82 (3)	2.13 (3)	2.928 (3)	164 (3)

Symmetry codes: (i) 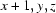; (ii) 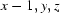; (iii) 
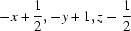
; (iv) 
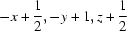
; (v) 
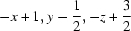
.

**Fig. 1.**
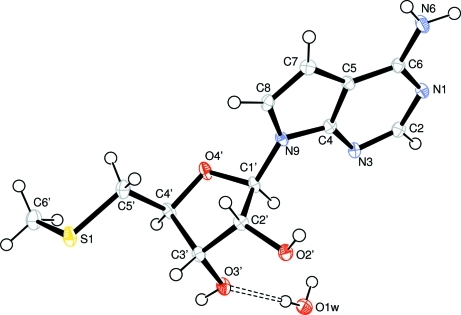
An ORTEP (Farrugia, 1999) view showing the asymmetric unit of (I) with 50% probabilility ellipsoids. The dotted lines represents an intermolecular hydrogen bond.

**Fig. 2.**
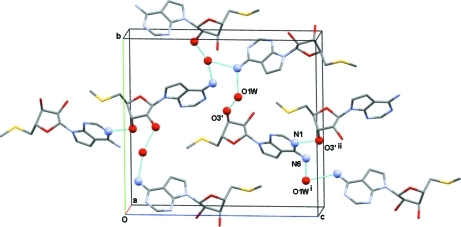
Mercury cell packing view (Macrae et al., 2006) emphasizing the links with the water of crystallization: conventional hydrogen bonds not running up the a axis are shown (dotted lines).For the complete hydrogen bonding set see Table 1. Contact atoms are shown in ball mode; H atoms are omitted for clarity. Symmetry operations: (i) 1 - x, y - 1/2, 3/2 - z (ii) 1/2 - x, 1 - y, 1/2 + z.

## supplementary crystallographic information

### Comment

The title compound was prepared as part of a study of purine transport or purine salvage pathway inhibitors with potential as alternative anti-malarial drugs (Riegelhaupt *et al.*, 2010). Its common name is 7-(5'Methylthio-β-D-erythrofuranosyl)-7*H*-pyrrolo[2,3-*d*]pyrimidin-2-amine monohydrate usually shortened to MT-tubercidin.H_2_O while the conventional name is 2-(4-Amino-pyrrolo[2,3-*d*]pyrimidin-7-yl)-5-methylsulfanylmethyl -tetrahydrofuran-3,4-diol monohydrate. The structural solution showed that in both batches there was an unexpected water molecule of crystallization, a likely cause of the variation in melting points with differently aged samples. The results for the better crystals are presented here. The asymmetric unit contents are shown in Figure 1.

The absolute configuration is defined as C1'(*R*), C2'(*R*), C3'(S) and C4'(S) with the ribofuranosyl unit adopting an (C2'-)*endo*-envelope Δ conformation (Q(2) 0.434 (3) Å, φ(2) 76.3 (3)°, Cremer & Pople (1975)). The 7*H*-pyrrolo[2,3-*d*]pyrimidine unit is very slightly puckered with total puckering amplitude of 0.035 (2) Å: its mean plane makes an angle of 88.40 (12)° with the mean plane through the ribofuranosyl unit. The orientation of the C4'–C5' bond is slightly different (with O4'-C1'-N9—C4 torsion angle of -126.6 (2)°) to that found in the related compounds 2'-Deoxy-2-fluorotubercidin (-110.2 (3)°, Seela *et al.*, 2007) and tubercidin (-112.8 (4)°, Abola & Sundaralingam, 1973). Other dimensions are normal.

The molecules are packed in three dimensions using 6 strong hydrogen bonds of the O–H···O and N–H···O types (Table 1). The graph set motifs (Etter *et al.*, 1990; Bernstein *et al.*, 1995) are extensive at the binary level: C^2^_2_(7), C^2^_2_(9), C^2^_2_(11), C^2^_2_(12), C^2^_2_(17), D^3^_3_(10), D^3^_3_(13), D^3^_3_(14), D^3^_3_(15), D^3^_3_(17) and D^3^_3_(18) types are found being based mainly on linked chains through the included water molecule (the latter feature is shown in Figure 2). The C–H···π interaction involving the methyl hydrogen H6'A and the 5-membered pyrrolo ring (at 2.80 Å) is considered fortuitous but noted here for completeness.

### Experimental

The title compound was prepared as described in the supplementary data section (compound 10) by Riegelhaupt *et al.* (2010).

### Refinement

The H atoms of the ordered hydroxyl, water and amine atoms were placed in the positions indicated by a difference electron density map and their positions were allowed to refine with *U*_iso_(H) = 1.5*U*_eq_(O,*N*). The water H atoms were restrained to an O–H distance of 0.82 (2) Å. The methyl H atoms were constrained to an ideal geometry (C—H = 0.98 Å) with *U*_iso_(H) = 1.5*U*_eq_(C), but were allowed to rotate freely about the adjacent C—C bonds. All other H atoms were placed in geometrically idealized positions and constrained to ride on their parent atoms with C—H distances of 0.95 (aromatic), 0.99 (methylene) or 1.00 (tertiary) Å with *U*_iso_(H) = 1.2*U*_eq_(C,*N*). Thirty-four high angle outlier reflections identified by having F_o_>>F_c_ and collected in the same area of reciprocal space (and with ΔF_o_**2/e.s.d. > 5) were omitted from the final cycles of refinement based on the lack of backstop mask corrections.

### Crystal data

**Table:**

C_12_H_16_N_4_O_3_S·H_2_O	*F*(000) = 664
*M**_r_* = 314.36	*D*_x_ = 1.456 Mg m^−^^3^
Orthorhombic, *P*2_1_2_1_2_1_	Cu *K*α radiation, λ = 1.54178 Å
Hall symbol: P 2ac 2ab	Cell parameters from 1322 reflections
*a* = 4.790 (1) Å	θ = 10.7–72.1°
*b* = 16.610 (3) Å	µ = 2.22 mm^−^^1^
*c* = 18.020 (4) Å	*T* = 100 K
*V* = 1433.7 (5) Å^3^	Needle, colourless
*Z* = 4	0.50 × 0.02 × 0.02 mm

### Data collection

**Table:**

Rigaku Spider diffractometer	2582 independent reflections
Radiation source: Rigaku MM007 rotating anode	2422 reflections with *I* > 2σ(*I*)
Rigaku VariMax-HF Confocal Optical System	*R*_int_ = 0.045
Detector resolution: 10 pixels mm^-1^	θ_max_ = 71.9°, θ_min_ = 7.3°
ω–scans	*h* = −5→2
Absorption correction: multi-scan (*ABSCOR*; Higashi, 1995)	*k* = −20→20
*T*_min_ = 0.712, *T*_max_ = 1.0	*l* = −21→19
8013 measured reflections	

### Refinement

**Table:**

Refinement on *F*^2^	Secondary atom site location: difference Fourier map
Least-squares matrix: full	Hydrogen site location: inferred from neighbouring sites
*R*[*F*^2^ > 2σ(*F*^2^)] = 0.039	H atoms treated by a mixture of independent and constrained refinement
*wR*(*F*^2^) = 0.091	*w* = 1/[σ^2^(*F*_o_^2^) + (0.031*P*)^2^ + 0.7234*P*] where *P* = (*F*_o_^2^ + 2*F*_c_^2^)/3
*S* = 1.08	(Δ/σ)_max_ < 0.001
2582 reflections	Δρ_max_ = 0.29 e Å^−^^3^
209 parameters	Δρ_min_ = −0.33 e Å^−^^3^
2 restraints	Absolute structure: Flack (1983), 986 Friedel pairs
Primary atom site location: structure-invariant direct methods	Flack parameter: 0.02 (2)

### Special details

**Table:**

Geometry. All e.s.d.'s (except the e.s.d. in the dihedral angle between two l.s. planes) are estimated using the full covariance matrix. The cell e.s.d.'s are taken into account individually in the estimation of e.s.d.'s in distances, angles and torsion angles; correlations between e.s.d.'s in cell parameters are only used when they are defined by crystal symmetry. An approximate (isotropic) treatment of cell e.s.d.'s is used for estimating e.s.d.'s involving l.s. planes.
Refinement. Refinement of *F*^2^ against ALL reflections. The weighted *R*-factor *wR* and goodness of fit *S* are based on *F*^2^, conventional *R*-factors *R* are based on *F*, with *F* set to zero for negative *F*^2^. The threshold expression of *F*^2^ > σ(*F*^2^) is used only for calculating *R*-factors(gt) *etc*. and is not relevant to the choice of reflections for refinement. *R*-factors based on *F*^2^ are statistically about twice as large as those based on *F*, and *R*- factors based on ALL data will be even larger.

### Fractional atomic coordinates and isotropic or equivalent isotropic displacement parameters (Å^2^)

**Table:**

	*x*	*y*	*z*	*U*_iso_*/*U*_eq_	
S1	0.97214 (14)	0.38389 (4)	0.36963 (3)	0.01889 (16)	
O1W	0.2297 (5)	0.66101 (10)	0.58560 (11)	0.0210 (4)	
H1A	0.363 (5)	0.6337 (16)	0.5723 (16)	0.032*	
H1B	0.110 (6)	0.6336 (16)	0.6041 (16)	0.032*	
O2'	0.8902 (4)	0.53482 (10)	0.64314 (9)	0.0155 (4)	
H2'O	0.971 (7)	0.5148 (17)	0.6768 (15)	0.023*	
O3'	0.6026 (4)	0.56178 (10)	0.51450 (10)	0.0171 (4)	
H3'O	0.553 (7)	0.5708 (17)	0.4704 (16)	0.026*	
O4'	0.4719 (4)	0.38417 (10)	0.55162 (8)	0.0166 (4)	
N1	0.1518 (5)	0.40830 (11)	0.87950 (11)	0.0148 (5)	
C2	0.0798 (6)	0.45356 (14)	0.82094 (13)	0.0152 (5)	
H2	−0.0681	0.4906	0.8293	0.018*	
N3	0.1866 (5)	0.45398 (11)	0.75258 (11)	0.0132 (4)	
C4	0.3891 (6)	0.39748 (13)	0.74463 (13)	0.0126 (5)	
C5	0.4795 (6)	0.34442 (13)	0.79930 (13)	0.0130 (5)	
C6	0.3551 (5)	0.35230 (14)	0.87012 (13)	0.0144 (5)	
N6	0.4318 (5)	0.30707 (13)	0.92757 (12)	0.0188 (5)	
H6A	0.355 (7)	0.3126 (17)	0.9726 (16)	0.028*	
H6B	0.552 (7)	0.2722 (17)	0.9214 (17)	0.028*	
C7	0.6937 (6)	0.29487 (14)	0.76725 (14)	0.0155 (5)	
H7	0.7949	0.2532	0.7913	0.019*	
C8	0.7228 (6)	0.31939 (14)	0.69593 (14)	0.0141 (5)	
H8	0.8501	0.2971	0.6611	0.017*	
N9	0.5374 (4)	0.38247 (11)	0.68120 (10)	0.0128 (4)	
C1'	0.5393 (6)	0.43227 (14)	0.61535 (12)	0.0139 (5)	
H1'	0.3971	0.4760	0.6208	0.017*	
C2'	0.8210 (6)	0.46963 (14)	0.59653 (13)	0.0133 (5)	
H2'	0.9698	0.4276	0.5998	0.016*	
C3'	0.7750 (6)	0.49214 (14)	0.51606 (14)	0.0138 (5)	
H3'	0.9552	0.5009	0.4892	0.017*	
C4'	0.6191 (6)	0.41680 (15)	0.48763 (13)	0.0139 (5)	
H4'	0.4815	0.4328	0.4486	0.017*	
C5'	0.8164 (6)	0.35258 (16)	0.45686 (13)	0.0188 (6)	
H5'A	0.7116	0.3019	0.4492	0.023*	
H5'B	0.9660	0.3419	0.4935	0.023*	
C6'	0.7305 (6)	0.34328 (16)	0.30226 (15)	0.0219 (6)	
H6'A	0.7358	0.2843	0.3041	0.033*	
H6'B	0.7835	0.3615	0.2525	0.033*	
H6'C	0.5412	0.3620	0.3137	0.033*	

### Atomic displacement parameters (Å^2^)

**Table:**

	*U*^11^	*U*^22^	*U*^33^	*U*^12^	*U*^13^	*U*^23^
S1	0.0145 (4)	0.0267 (3)	0.0155 (3)	−0.0023 (3)	0.0037 (3)	−0.0050 (3)
O1W	0.0206 (12)	0.0217 (9)	0.0208 (10)	−0.0034 (8)	0.0045 (8)	0.0000 (8)
O2'	0.0170 (10)	0.0174 (8)	0.0120 (9)	−0.0004 (7)	−0.0028 (8)	−0.0007 (7)
O3'	0.0200 (11)	0.0205 (9)	0.0108 (8)	0.0075 (8)	−0.0027 (8)	0.0023 (7)
O4'	0.0138 (10)	0.0262 (9)	0.0096 (8)	−0.0044 (9)	0.0006 (8)	−0.0020 (7)
N1	0.0160 (12)	0.0172 (10)	0.0113 (10)	0.0002 (8)	−0.0001 (9)	0.0006 (8)
C2	0.0135 (14)	0.0164 (11)	0.0157 (12)	0.0007 (10)	−0.0020 (11)	−0.0023 (10)
N3	0.0108 (11)	0.0172 (9)	0.0117 (11)	0.0010 (8)	−0.0005 (9)	−0.0015 (9)
C4	0.0102 (13)	0.0155 (11)	0.0121 (12)	−0.0032 (9)	−0.0034 (10)	−0.0014 (10)
C5	0.0113 (14)	0.0155 (10)	0.0123 (11)	−0.0002 (10)	0.0009 (11)	0.0012 (9)
C6	0.0153 (14)	0.0157 (11)	0.0121 (11)	−0.0018 (10)	−0.0028 (11)	−0.0004 (10)
N6	0.0235 (15)	0.0226 (10)	0.0104 (10)	0.0066 (10)	0.0018 (10)	0.0015 (9)
C7	0.0130 (14)	0.0162 (11)	0.0174 (12)	0.0012 (10)	−0.0002 (12)	0.0008 (10)
C8	0.0105 (14)	0.0158 (11)	0.0160 (13)	0.0017 (10)	0.0025 (11)	−0.0035 (10)
N9	0.0104 (11)	0.0170 (9)	0.0110 (10)	−0.0010 (9)	0.0025 (9)	0.0006 (8)
C1'	0.0133 (15)	0.0196 (11)	0.0089 (11)	0.0001 (10)	−0.0008 (10)	−0.0001 (9)
C2'	0.0077 (13)	0.0178 (11)	0.0142 (12)	−0.0002 (10)	−0.0020 (11)	0.0008 (10)
C3'	0.0110 (14)	0.0174 (11)	0.0131 (12)	0.0020 (10)	0.0011 (11)	0.0001 (10)
C4'	0.0098 (14)	0.0227 (12)	0.0091 (11)	0.0009 (10)	−0.0027 (10)	0.0029 (10)
C5'	0.0184 (15)	0.0227 (12)	0.0153 (12)	0.0002 (12)	−0.0011 (12)	−0.0042 (11)
C6'	0.0177 (16)	0.0283 (14)	0.0197 (14)	−0.0005 (12)	−0.0007 (12)	−0.0054 (12)

### Geometric parameters (Å, °)

**Table:**

S1—C6'	1.808 (3)	N6—H6A	0.90 (3)
S1—C5'	1.816 (3)	N6—H6B	0.82 (3)
O1W—H1A	0.820 (18)	C7—C8	1.355 (3)
O1W—H1B	0.805 (18)	C7—H7	0.9500
O2'—C2'	1.410 (3)	C8—N9	1.399 (3)
O2'—H2'O	0.79 (3)	C8—H8	0.9500
O3'—C3'	1.422 (3)	N9—C1'	1.446 (3)
O3'—H3'O	0.84 (3)	C1'—C2'	1.523 (4)
O4'—C1'	1.436 (3)	C1'—H1'	1.0000
O4'—C4'	1.456 (3)	C2'—C3'	1.514 (3)
N1—C2	1.341 (3)	C2'—H2'	1.0000
N1—C6	1.357 (3)	C3'—C4'	1.545 (3)
C2—N3	1.334 (3)	C3'—H3'	1.0000
C2—H2	0.9500	C4'—C5'	1.529 (3)
N3—C4	1.357 (3)	C4'—H4'	1.0000
C4—N9	1.369 (3)	C5'—H5'A	0.9900
C4—C5	1.391 (3)	C5'—H5'B	0.9900
C5—C6	1.415 (3)	C6'—H6'A	0.9800
C5—C7	1.436 (3)	C6'—H6'B	0.9800
C6—N6	1.331 (3)	C6'—H6'C	0.9800
			
C6'—S1—C5'	102.20 (14)	O4'—C1'—H1'	109.2
H1A—O1W—H1B	111 (3)	N9—C1'—H1'	109.2
C2'—O2'—H2'O	104 (2)	C2'—C1'—H1'	109.2
C3'—O3'—H3'O	109 (2)	O2'—C2'—C3'	114.53 (19)
C1'—O4'—C4'	108.50 (17)	O2'—C2'—C1'	112.9 (2)
C2—N1—C6	118.1 (2)	C3'—C2'—C1'	100.7 (2)
N3—C2—N1	129.2 (2)	O2'—C2'—H2'	109.5
N3—C2—H2	115.4	C3'—C2'—H2'	109.5
N1—C2—H2	115.4	C1'—C2'—H2'	109.5
C2—N3—C4	111.6 (2)	O3'—C3'—C2'	107.7 (2)
N3—C4—N9	125.8 (2)	O3'—C3'—C4'	111.8 (2)
N3—C4—C5	125.9 (2)	C2'—C3'—C4'	100.83 (19)
N9—C4—C5	108.3 (2)	O3'—C3'—H3'	112.0
C4—C5—C6	116.7 (2)	C2'—C3'—H3'	112.0
C4—C5—C7	107.5 (2)	C4'—C3'—H3'	112.0
C6—C5—C7	135.8 (2)	O4'—C4'—C5'	109.07 (19)
N6—C6—N1	119.2 (2)	O4'—C4'—C3'	105.84 (19)
N6—C6—C5	122.2 (2)	C5'—C4'—C3'	112.7 (2)
N1—C6—C5	118.6 (2)	O4'—C4'—H4'	109.7
C6—N6—H6A	122 (2)	C5'—C4'—H4'	109.7
C6—N6—H6B	119 (2)	C3'—C4'—H4'	109.7
H6A—N6—H6B	119 (3)	C4'—C5'—S1	111.59 (18)
C8—C7—C5	106.4 (2)	C4'—C5'—H5'A	109.3
C8—C7—H7	126.8	S1—C5'—H5'A	109.3
C5—C7—H7	126.8	C4'—C5'—H5'B	109.3
C7—C8—N9	109.9 (2)	S1—C5'—H5'B	109.3
C7—C8—H8	125.1	H5'A—C5'—H5'B	108.0
N9—C8—H8	125.1	S1—C6'—H6'A	109.5
C4—N9—C8	107.91 (19)	S1—C6'—H6'B	109.5
C4—N9—C1'	125.7 (2)	H6'A—C6'—H6'B	109.5
C8—N9—C1'	125.5 (2)	S1—C6'—H6'C	109.5
O4'—C1'—N9	109.66 (18)	H6'A—C6'—H6'C	109.5
O4'—C1'—C2'	104.34 (19)	H6'B—C6'—H6'C	109.5
N9—C1'—C2'	114.9 (2)		
			
C6—N1—C2—N3	−2.3 (4)	C4'—O4'—C1'—N9	−148.2 (2)
N1—C2—N3—C4	2.3 (4)	C4'—O4'—C1'—C2'	−24.6 (2)
C2—N3—C4—N9	179.6 (2)	C4—N9—C1'—O4'	−126.6 (2)
C2—N3—C4—C5	0.3 (3)	C8—N9—C1'—O4'	65.6 (3)
N3—C4—C5—C6	−2.4 (4)	C4—N9—C1'—C2'	116.2 (3)
N9—C4—C5—C6	178.2 (2)	C8—N9—C1'—C2'	−51.6 (3)
N3—C4—C5—C7	179.5 (2)	O4'—C1'—C2'—O2'	164.56 (18)
N9—C4—C5—C7	0.1 (3)	N9—C1'—C2'—O2'	−75.3 (3)
C2—N1—C6—N6	179.5 (2)	O4'—C1'—C2'—C3'	42.0 (2)
C2—N1—C6—C5	−0.2 (3)	N9—C1'—C2'—C3'	162.11 (19)
C4—C5—C6—N6	−177.4 (2)	O2'—C2'—C3'—O3'	−45.9 (3)
C7—C5—C6—N6	0.1 (5)	C1'—C2'—C3'—O3'	75.5 (2)
C4—C5—C6—N1	2.2 (3)	O2'—C2'—C3'—C4'	−163.2 (2)
C7—C5—C6—N1	179.7 (3)	C1'—C2'—C3'—C4'	−41.8 (2)
C4—C5—C7—C8	−0.1 (3)	C1'—O4'—C4'—C5'	119.1 (2)
C6—C5—C7—C8	−177.8 (3)	C1'—O4'—C4'—C3'	−2.5 (2)
C5—C7—C8—N9	0.2 (3)	O3'—C3'—C4'—O4'	−85.9 (2)
N3—C4—N9—C8	−179.4 (2)	C2'—C3'—C4'—O4'	28.4 (2)
C5—C4—N9—C8	0.0 (3)	O3'—C3'—C4'—C5'	155.0 (2)
N3—C4—N9—C1'	11.0 (4)	C2'—C3'—C4'—C5'	−90.8 (2)
C5—C4—N9—C1'	−169.5 (2)	O4'—C4'—C5'—S1	172.20 (16)
C7—C8—N9—C4	−0.1 (3)	C3'—C4'—C5'—S1	−70.5 (2)
C7—C8—N9—C1'	169.5 (2)	C6'—S1—C5'—C4'	−91.9 (2)

### Hydrogen-bond geometry (Å, °)

**Table:**

*D*—H···*A*	*D*—H	H···*A*	*D*···*A*	*D*—H···*A*
O2'—H2'O···N3^i^	0.79 (3)	1.99 (3)	2.776 (3)	173 (3)
O1W—H1A···O3'	0.82 (3)	1.96 (3)	2.748 (3)	162 (3)
O1W—H1B···O2'^ii^	0.80 (3)	2.07 (3)	2.848 (3)	162 (3)
O3'—H3'O···N1^iii^	0.84 (3)	1.94 (3)	2.766 (3)	166 (3)
N6—H6A···O1W^iv^	0.90 (3)	2.12 (3)	2.998 (3)	166 (3)
N6—H6B···O1W^v^	0.82 (3)	2.13 (3)	2.928 (3)	164 (3)

Symmetry codes: (i) *x*+1, *y*, *z*; (ii) *x*−1, *y*, *z*; (iii) −*x*+1/2, −*y*+1, *z*−1/2; (iv) −*x*+1/2, −*y*+1, *z*+1/2; (v) −*x*+1, *y*−1/2, −*z*+3/2.
